# Evaluating the effects of curcumin nanomicelles on clinical outcome and cellular immune responses in critically ill sepsis patients: A randomized, double-blind, and placebo-controlled trial

**DOI:** 10.3389/fnut.2022.1037861

**Published:** 2022-12-06

**Authors:** Arash Karimi, Sanaz Pourreza, Mahdi Vajdi, Ata Mahmoodpoor, Sarvin Sanaie, Mozhde Karimi, Ali Tarighat-Esfanjani

**Affiliations:** ^1^Department of Clinical Nutrition, Faculty of Nutrition and Food Sciences, Tabriz University of Medical Sciences, Tabriz, Iran; ^2^Nutrition Research Center, Faculty of Nutrition and Food Sciences, Tabriz University of Medical Sciences, Tabriz, Iran; ^3^Department of Community Nutrition, School of Nutritional Sciences and Dietetics, Tehran University of Medical Sciences, Tehran, Iran; ^4^Student Research Committee, Department of Clinical Nutrition, School of Nutrition and Food Sciences, Isfahan University of Medical Sciences, Isfahan, Iran; ^5^Department of Anesthesiology and Intensive Care, Faculty of Medicine, Tabriz University of Medical Sciences, Tabriz, Iran; ^6^Research Center for Integrative Medicine in Aging, Aging Research Institute, Tabriz University of Medical Sciences, Tabriz, Iran; ^7^Department of Immunology, School of Medicine, Tarbiat Modares University, Tehran, Iran

**Keywords:** curcumin, immune response, clinical outcome, sepsis, inflammation

## Abstract

**Introduction:**

In sepsis, the immune system is overreacting to infection, leading to organ dysfunction and death. The purpose of this study was to investigate the impacts of curcumin nanomicelles on clinical outcomes and cellular immune responses in critically ill sepsis patients.

**Method:**

For 10 days, 40 patients in the intensive care units (ICU) were randomized between the nano curcumin (NC) and placebo groups in a randomized study. We evaluated serum levels of biochemical factors, inflammatory biomarkers, the mRNA expression levels of FOXP3, NLRP-3, IFN-γ, and NF-κp genes in the PBMCs, and clinical outcomes before the beginning of the supplementation and on days 5 and 10.

**Results:**

NLR family pyrin domain containing 3 (NLRP3), interferon gamma (IFN-γ), and nuclear factor kappa-light-chain-enhancer of activated B cells (NF-κB) mRNA expression levels significantly *P* = 0.014, *P* = 0.014, and *P* = 0.019, respectively) decreased, but forkhead box P3 (FOXP3) mRNA expression levels increased significantly (*P* = 0.008) in the NC group compared to the placebo group after 10 days. NC supplementation decreased serum levels of IL-22, IL-17, and high mobility group box 1 (HMGB1) (*P* < 0.05). Nevertheless, biochemical factors and nutritional status did not differ significantly (*P* > 0.05). NC supplementation resulted in decreased sequential organ failure assessment and multiple organ dysfunction syndromes scores, while it did not have significant impacts on length of stay in the ICU, systolic blood pressure, diastolic blood pressure, a saturation of oxygen (%), and respiratory rate (breaths/min) PaO2/FiO2 (*p* > 0.05).

**Conclusion:**

For critically ill patients with sepsis, NC supplementation may be an effective therapeutic strategy. More randomized clinical trials involving longer follow-up periods and different doses are needed to achieve the best results.

## Introduction

Sepsis is a complex and severe disorder that is caused by a strong response of the body’s immune system to an infection. The disease is by far the most serious medical problem related to acute organ dysfunction and the high hazard of death in the intensive care unit (ICU) (1). The immune system’s excessive response leads to an enhancement in inflammatory oxidative stress and an increase in organ failure (2, 3). In the world, disease continues to be the leading cause of death (2, 3). Globally, sepsis affects an estimated 30 million people worldwide, and this number has increased annually by nine to 13 percent. According to global statistics, sepsis affected 48.9 million people worldwide in 2017 and caused 11.0 million deaths (4).

There are two parts to the human immune system: innate and adaptive (5). The innate immune system responds non-specifically to infections (6). Adaptive immunity is slower than innate immunity but may recognize unique antigens and establish immunity after multiple exposures. Innate system cells include basophils, mast cells, eosinophils, natural killer (NK) cells, dendritic cells, macrophages, and neutrophils (7). B and T cells comprise the adaptive immune system responding to pathogens (8). B cells generate antibodies and plasma cells for long-term immunity, whereas T cells, namely gamma delta (γδ), CD8+, CD4+, and regulatory T cells (Tregs), create plasma cells and antibodies for long-term immunity (9). In addition to activating immune responses, the entry of pathogens into the body leads to the activation of various inflammatory pathways, namely NLRs, HMGB-1, and NF-κB (10). The NLRs play a significant role in recognizing invading bacteria and initiating the innate immune response. Inflammasomes can be activated during sepsis to augment inflammatory responses (11). As a result of NLRP3 inflammasome activation, caspase-1 is activated, producing pro-inflammatory cytokines, IL-18, and IL-1β (12).

On the other hand, the activation of NLRP3 leads to up-regulated NF-κB pathway, which can cause the production of various swelling factors. The nuclear protein HMGB1 regulates innate immune responses both intracellularly and extracellularly and is found ubiquitously in almost all cells (13). HMGB1 also functions as an acute-phase cytokine during infection. Serum and tissue HMGB1 levels rise during infection, particularly in sepsis, and play a crucial role in systemic inflammation (14). On the other hand, the enhancement in the serum level of inflammatory cytokines causes a decrease in the level of albumin, urea, and BUN bilirubin, LDH, in sepsis patients (15). Impaired nutritional variables (energy intake and serum albumin) are expected to exacerbate clinical outcomes, namely sequential organ failure assessment (SOFA) and multiple organ dysfunction syndromes (MODS) score, PaO2/FiO2, duration of mechanical ventilation, and duration of ICU stay (16). In addition, sepsis is associated with an excessive reduction of forkhead box P3 (FOXP3) (12). FOXP3 regulates the development and activity of CD25^+^ CD4^+^ Treg cells, which play an important role in the immune response (12).

Notwithstanding the complexity of sepsis in people admitted to the ICU, a variety of treatments, such as corticosteroids and broad-spectrum antibiotics, are used today, but efforts to find effective treatment with minimal side effects are still ongoing (17). Accordingly, various studies indicate that natural immunomodulatory agents might ameliorate bacterial and virus diseases when combined with routine treatment (18). Rolta et al. (19), Rolta et al. (20) showed that herbal compounds can be used as an adjunctive treatment in corona. In other studies Rolta et al. (21) phytocompounds indicated (emodin, rhein13c6, chrysophenol dimethyl ether and resveratrol) have antibacterial and antifungal properties.

A hydrophobic polyphenol and an active component in turmeric is curcumin, derived from Curcuma longa rhizomes. Curcuminoids are comprised of three components, including bisdemethoxycurcumin (10 to 15%), demethoxycurcumin (20 to 27%), and curcumin (60 to 70%) (22). Numerous pieces of evidence show that curcumin has many pharmacological and therapeutic activities, including antimicrobial, antioxidant, and antiviral effects, anti-cancer, and anti-inflammatory (23). Curcumin works by targeting multiple biochemical pathways, such as reducing lipid peroxidation, increasing the expression of antioxidant-producing genes, attenuating NF-κB and NRLP3 signaling pathways, and, most importantly, modulating the immune system response (24). Although curcumin has many medicinal benefits, it is unfortunately absorbed in very small amounts due to its low bioavailability and rapid metabolism, which is exacerbated in patients admitted to the ICU (23). However, today, through effective methods such as the use of liposomes, the use of nanoparticles, including non-polar sandwich technology, nano micelles, complexing with phospholipids or piperine and solid lipid particle formulations leads to a substantial rise in the absorption of hydrophobic substances such as curcumin (25). Also, due to the proven advantageous impacts of curcumin in cell lines and on models of septic rats, as well as some human studies on sepsis (24), this randomized clinical trial (RCT) aims to the effects of NC on immune system responses and clinical outcomes in critically ill patients with sepsis.

## Materials and methods

### Study design

We conducted this study on the 40 hospitalized patients in the ICUs at Imam Reza and Shohada Hospitals (Tabriz University of Medical Sciences, Tabriz, Iran). Criteria for inclusion included critically ill patients feeding enteral nutrition and patients who had been in the ICU for at least 10 days. Furthermore, An exclusion criteria included participants with the following conditions: patients with intestinal ischemia, pancreatitis, intolerance to enteral feeding, short bowel syndrome, pregnant and lactating women, intestinal obstruction, and use of non-steroidal anti-inflammatory drugs (NSAIDs). Researchers registered the study at the Iranian Registry of Clinical Trials (IRCT) website (IRCT20110123005670N7). A nosocomial infection is defined by the most recent guidelines of the CDC (26).

### Randomization and intervention

Patients in blocks arranged according to gender and age score were randomly divided into placebo or NC groups in a 1:1 ratio using RAS software. Both patients and researchers were blind to the allocation of the study. Patients in the supplementation group received routine therapy, namely antibiotics (Meropenem, Imipenem, Ciprofloxacin) with two 80 mg NC capsules, while the placebo group received routine therapy with a placebo for 10 days. Enteral feedings were administered through the nasogastric tube to all patients from their first 24 h of admission (Karen Company, Tehran, Iran; Table 1). Depending on each patient’s metabolic status and weight, the amount of energy required was calculated at 25 to 30 kcal/kg. Starting with 25 ml/h of enteral feeding, the rate was enhanced by 25 ml/h every 4 h until the aim rate was reached. In cases where the gastric residual volume exceeded 150 ml, prokinetic agents were administered. Exir-Nano-Sina company produced a placebo and NC capsules (batch number: 17003). In NC supplements, curcumin accounted for bis-desmethoxycurcumin for 3%, for 25%, and 72%, emethoxycurcumin. The NC formulation included polysorbate 80 as a component of the placebo capsules. A specialist evaluated patients according to inclusion criteria before enrolling them in the study. Nurses without knowing which is the placebo and the NC, every 12 h (9:00 a.m. and 9:00 p.m.), an hour later than enteral feeding (to prevent interaction with the contents of the formula received), NC capsules and placebo (in terms of form and size) were given to patients as a solution through a nasogastric tube.

**TABLE 1:** Contents of patients enteral formulas.

Nutrient	Amount per 11.3 g	%DV	Amount per 1000 ml solution	Nutrient	Amount per 11.3 g	%DV	Amount per 1000 ml solution
Calories (Kcal)	50		1000	Biotin (mcg)	8.2	3	163.7
Protein (g)	1.8	3.6	36	Calcium (mg)	28.15	3	563
Total carbohydrate (g)	6.76	2.2	135	Chromium (mcg)	1	0.8	19.2
Dietary Fiber (g)	0.23	0.9	4.4	Copper (mcg)	42	2	851
Total Fat (g)	1.8	3	36	Fluoride (mg)	0.1	–	2.2
Vitamin A (IU)	108	2	2160	Iodine (mcg)	4.9	3.3	98.2
Vitamin D (IU)	9.8	2.5	196.4	Iron (mg)	0.48	2.7	9.5
Vitamin E (IU)	1.3	4	25.6	Magnesium (mg)	12.2	3	243.7
Vitamin K (mcg)	3.3	4	65.3	Manganese (mg)	0.08	4	1.6
Vitamin C (mg)	4.9	8	98.2	Molybdenum (mcg)	1.5	2	29.5
Vitamin B1 (mg)	0.08	5	1.6	Phosphorus (mg)	27	2.7	530
Vitamin B2 (mg)	0.09	5	1.7	Zinc (mg)	0.48	3	9.7
Niacin (mg)	0.65	3	13	Selenium (mcg)	1.8	2.6	3.6
Vitamin B6 (mg)	0.07	3	1.35	Sodium (mg)	50.7	2	1013.4
Folic acid (mcg)	13.1	3	261.9	Potassium (mg)	73.2	2	1435.4
Vitamin B12 (mcg)	0.26	4	5.1	Chloride (mg)	42.8	1.2	855.7
Pantothenic Acid (mg)	0.3	3	6.5	L- Carnitine (mg)	4.5	–	90.1

Since the vast majority of patients with sepsis have a low Glasgow Coma Score, in this study, we obtained informed consent from first-degree (but legal) relatives such as mothers, fathers, sons, or daughters of patients before entering the study.

### Laboratory evaluates

Before the intervention, 5th, and 10th, every day between 12:00 and 3:00 p.m., venous blood samples were taken from each patient. The biochemical factors, namely blood urea nitrogen (BUN), albumin, fasting blood sugar (FBS), hemoglobin, indirect bilirubin, direct bilirubin, lactate dehydrogenase (LDH) and total plasma protein, were specified using Abbott ALCYON-350 auto-analyzer kits. The blood samples of patients were centrifuged for 10 min at a speed of 2500 rpm (Beckman Avanti J-25 - Beckman Coulter, Brea, CA). The serum was stored at 70^°^C before biochemical assessments. According to dual biotin antibody sandwich technology, inflammatory markers (IL-17 and IL-22) were assessed using the enzyme-linked immunoassay (ELISA) method. In the present study, Human IL-17 and IL-22 ELISA kits made by the Assessment Technology Laboratory (Crystal Day Biotech Co., Ltd., Shanghai, China) and HMGB1 were used.

### Peripheral blood mononuclear cells and RNA isolation

Whole blood samples were directly examined for isolation of peripheral blood mononuclear cells (PBMCs). Separating PBMCs by density gradient centrifugation was accomplished using Ficoll-Histopaque solution gradient centrifugation. To isolate total RNA from the blood, TRIzol was used (Sigma Aldrich, Germany). Quantitative and qualitative characteristics of extracted RNA were determined using a NanoDrop spectrophotometer (Nano-Drop One/Once, Thermo Scientific). Then, we performed reverse transcription with random hexamer primers and oligo (dT) to transform the total RNA into complementary DNA (cDNA) based on the producer’s instructions (BioFact, RTase, South Korea). Gel electrophoresis on 1% agarose gel was used to determine the RNA integration.

### Real-time polymerase chain reaction for genes

Measuring the levels of mRNA expression levels of FOXP3, NLRP3, IFN-γ, and NF-kβ was done using real-time polymerase chain reaction (RT-PCR) (Sigma Aldrich, Germany). The manufacturer’s instructions were followed for the reverse transcription of 25 ng of total RNA and for constructing complementary DNA using reverse transcription reagent kits (Thermo Scientific, EU). With the Light-Cycler 480 instrument (Roche, Germany), qRT-PCR was performed in a volume of 10 μl using SYBR Green PCR Master Mix (Sigma-Aldrich, Germany). A three-phase thermal cycling procedure was conducted: phase one (primary denaturation: 95^°^C for two min), phase two (30 s at 63^°^C, and 30 s at 74^°^C, 34 to 42 cycles of 30 s at 96^°^C), and last phase to form the melt curve (5 min at 74^°^C). A Primer Bank sequence was used to design the primers. The characteristic primers for the human of FOXP3, NLRP-3, IFN-γ NF-kβ, and β-actin genes are resumed in Table 2. Using the 2^–ΔΔCT^ method as the comparison of placebo/post-intervention, the relative expression levels for each gene were calculated for each reaction in triplicate.

**TABLE 2:** Sequence of gene primers for qRT-PCR.

Genes	Forward and reverse	Sequences
FOXP3	F R	5′-TTCAGCCAGCCCAGCACATC-3′ 5′-CGTAGCCGAAGAAACCTCATTGTC-3′
NLRP-3	F R	5′-ATGTGTGTGGAGAGCGTCAACC-3′ 5′-TGAGCAGAGTCTTCAGAGACAGCC-3′
IFN-γ	F R	5′-CCTTTTCTACTTTGCCAGCAAAC-3′ 5′-GAGGCCGTCCCAACCAC-3′
NF-kβ	F R	5′-TCCCTGAACCCTATGAAC-3′ 5′-CTAAACCAGCCAGACCTT-3′
β-Actin	F R	5′-GAGCTACGAGCTGCCTGACG-3′ 5′-GTAGTTTCGTGGATGCCACAG-3′

### Statistical analysis

In this study, sampling based on the standard equation (Pukak) and based on mean and standard deviation with a significance level test of 5% (α = 0.05) with considering 80% (β = 0.2) power and distance 95% confidence was performed. According to the method for calculating the sample size for clinical trials, each group’s sample size was 17 individuals (27). In addition, 20% of dropouts were taken into account, increasing this to 20 people. The data was analyzed using SPSS software version 24 (Chicago, IL, USA). For the assessment of the normal distribution of continuous variables, the Kolmogorov-Smirnov test was used. Quantitative data are presented as frequency (%). For normally distributed data, the means and standard deviations (SDs) are shown as a mean ± standard deviation; for non-normally distributed data, the Q1 and Q3. We used Mann–Whitney U, independent *t*-tests, and chi-square tests to compare group changes (endpoint minus baseline). A paired *t*-test was used to determine if there were significant differences between baseline and after the intervention. Analyzing covariance (ANCOVA) was used to eliminate confounding variables and examine differences between post-intervention groups.

## Results

### Characteristics of patients participating

**FIGURE 1:**
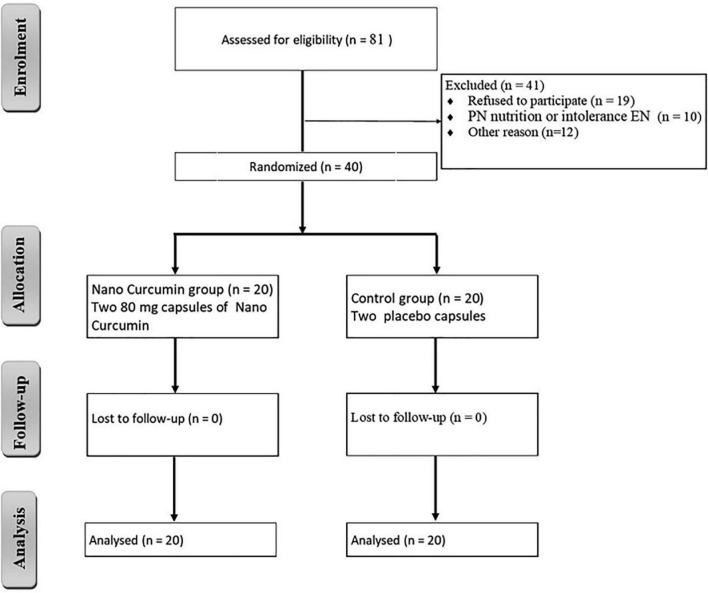
Study flow diagram.

In this clinical study, 81 patients were included in the study. Also, 41 patients were excluded from the study due to discharge, refusal to participate, and intolerance to enteral nutrition. A total of 20 patients in the NC group and 20 patients in the placebo group participated in the current study, as shown in Figure 1. Table 3 summarizes the demographic data of participants. The baseline characteristics of the participants did not significantly differ between the two groups.

**TABLE 3:** Summary of baseline characteristics of the patients.

Variables	Nano-curcumin group (*n* = 20)	Placebo group (*n* = 20)	*Pv*
Age, yrs	48.75 ± 7.19	49.10 ± 5.01	0.057^¥^
**Sex, *n* (%)**			
Males	11(55)	13(65)	0.257^†^
Females	9 (45)	7(35)	
Body Temperature (°C)	38.52 ± 0.87	38.39 ± 0.61	0.874^¥^
Heart rate (beats/min)	99.64 ± 22.74	97.51 ± 23.14	0.613^¥^
Respiratory rate (breaths/min)	18 (15–23)	18 (16–23)	0.911^§^
WBC (10^9^/L)	14.30 ± 2.15	13.02 ± 2.59	0.828^¥^
Lactate (mmol/L)	6.89 ± 4.6	6.16 ± 3.9	0.716^¥^
Na^+^ (mmol/L)	146.16 ± 4.87	148.66 ± 5.01	0.531^¥^
K^+^ (mmol/L)	4.51 ± 0.88	4.49 ± 0.76	0.767^¥^
APACH II score	17 (13–21)	16.60 (13–19)	0.231^§^
SOFA score	8.10 (5–11)	7.50 (6–11)	0.538§
**Reason for ICU admission, *n*(%)**			
Medical	4 (20)	5(25)	0.487^†^
Surgical, Trauma	12 (80)	14 (75)	
Energy requirements (Kcal/day)	2059.51 ± 153.27	1979.23 ± 133.98	0.614^¥^
Energy intake (Kcal/day)	1445.74 ± 149.90	1462.45 ± 132.24	0.231^¥^
**Source of sepsis**			
Pulmonary infection (*n*,%)	7 (35)	6 (30)	0.698^†^
Abdominal infection (*n*,%)	6 (30)	5 (25)	
Urinary tract infection (*n*,%)	3 (15)	3 (15)	
Bacteriemia (*n*,%)	4 (20)	6 (30)	

APACHE, acute physiology and chronic health evaluation; SOFA, sequential organ failure assessment, ICU, intensive care unit; WBC, white blood cell.

Data are presented as mean ± SD or number (%).

^¥^Based on independent sample *t*-test.

^†^Based on Pearson’s chi-squared test.

^§^Based on Mann–Whitney U Test.

### Effect of nano curcumin on nutritional status

Between the NC group and placebo group, there were no considerable alterations in energy intake during the study period, as shown in Table 4.

**TABLE 4:** Changes nutritional status during the study.

Variables		Nano-curcumin group (*n* = 20)	Placebo group (*n* = 20)	*Pv*
Energy requirements (kcal/day)	Day 0 Day 10 P^†^	2059.51 ± 153.27 2487.05 ± 169.1 **0.002**	1979.23 ± 133.98 2296.87 ± 115.30 **0.034**	0.614^¥^ 0.736^$^
Mean caloric intake (kcal/day)	Day 0 Day 10 P^†^	1445.74 ± 149.90 1721.65 ± 146.82 **<0.0001**	1462.45 ± 132.24 1691.97 ± 146.71 **<0.0001**	0.718 0.232^$^

Data are presented as means ± SD; Numbers in bold are statistically significant *p*-value < 0.05.

^†^Based on paired *t*-test.

^¥^Based on independent sample *t*-test.

^$^Based on analysis of covariance (ANCOVA); adjusted for sex, BMI, age, standard treatment, type of disease and baseline values.

^α^Based on Mann–Whitney U Test.

### Effect of nano curcumin on biochemical factors

In Table 5 the effect of curcumin on biochemical factors during the study stages is shown. Albeit serum levels of BUN, FBS, albumin, hemoglobin, total bilirubin, direct bilirubin, lactate, and total plasma protein decreased significantly in the NC group, the intergroup changes were not statistically significant.

**TABLE 5:** Effect of curcumin on biochemical factors during the study stages.

Variables		Nano-curcumin group (*n* = 20)	Placebo group (*n* = 20)	*Pv*
BUN (mg/dL)	Day 0 Day 5 Day 10 P^‡^	71.6 ± 20.8 62.6 ± 18.92 50.6 ± 17.26 **0.012**	67.32 ± 18.36 59.56 ± 17.15 49.06 ± 15.74 **0.026**	0.453^¥^ 0.323^¥^ 0.501^£^
Albumin (g/dL)	Day 0 Day 5 Day 10 P^‡^	3.16 ± 0.62 3.34 ± 0.52 3.86 ± 0.70 0.011	3.12 ± 0.58 3.36 ± 0.14 3.59 ± 0.61 0.039	0.699^¥^ 0.739 ^£^ 0.314^£^
FBS (mmol/L)	Day 0 Day 5 Day 10 P^‡^	130.53 ± 51.33 126.76 ± 40.16 121.27 ± 29.33 **0.063**	132.91 ± 39.17 129.85 ± 26.64 125.12 ± 51.33 0.116	0.721^¥^ 0.564 ^£^ 0.237^£^
Hemoglobin, (g/dL)	Day 0 Day 5 Day 10 P^‡^	15.23 ± 2.89 14.15 ± 2.43 12.89 ± 1.98 **0.035**	14.83 ± 3.03 13.75 ± 2.72 12.36 ± 2.10 0.063	0.465^¥^ 0.369 ^£^ 0.212^£^
Indirect bilirubin, (mg/dL)	Day 0 Day 5 Day 10 P^‡^	1.01 ± 0.25 09.34 ± 0.14 07.86 ± 0.23 **0.036**	1.23 ± 0.31 09.75 ± 0.21 08.26 ± 0.38 **0.075**	0.704^¥^ 0.520^£^ 0.281^£^
Direct bilirubin, (mg/dL)	Day 0 Day 5 Day 10 P^‡^	06.21 ± 0.07 05.11 ± 0.04 03.41 ± 0.03 **0.041**	07.01 ± 0.07 05.81 ± 0.04 04.01 ± 0.08 0.069	0.408^¥^ 0.616 ^£^ 0.225^£^
LDH (mg/dL)	Day 0 Day 5 Day 10 P^‡^	20.61 ± 4.6 18.24 ± 3.26 15.89 ± 2.79	19.84 ± 3.9 17.63 ± 3.06 16.03 ± 2.53	0.510^¥^ 0.411 ^£^ 0.140^£^
Total plasma protein, (mg/dL)	Day 0 Day 5 Day 10 P^‡^	101.63 ± 9.07 98.71 ± 3.01 84.12 ± 2.58 **0.011**	114.08 ± 10.24 100.98 ± 3.01 90.42 ± 2.58 **0.045**	0.617^¥^ 0.821 ^£^ 0.459^£^

FBS, fasting blood sugar; LDH: lactate dehydrogenase; BUN: blood urea nitrogen.

Data are presented as means ± SD; Numbers in bold are statistically significant *p*-value < 0.05.

^¥^Based on independent sample *t*-test.

^£^Based on analysis of covariance (ANCOVA); adjusted for sex, BMI, age, standard treatment, type of disease and baseline values.

^‡^Based on repeated-measure analysis of variance.

### Effect of nano curcumin on inflammatory factors

In Table 6, the effect of nano curcumin supplementation on inflammatory factors during the study stages is shown. There were no considerable alterations between the study groups in the baseline serum levels of HMGB-1, IL-17, and IL-22, however, compared to the placebo group, HMGB-1, IL-17, and IL-22 serum concentrations were significantly lower in the NC group after 10 days.

**TABLE 6:** Effect of nano curcumin supplementation on inflammatory factors.

Variables		Nano curcumin group (*n* = 20)	Placebo group (*n* = 20)	*Pv*
HMGB-1	Day 0 Day 5 Day 10 P^‡^	13.32 ± 2.02 11.05 ± 1.73 7.86 ± 1.34 0.016	15.48 ± 2.82 13.56 ± 1.94 11.59 ± 1.53 0.384	0.515^¥^ 0.754 ^£^ 0.006^£^
IL-17	Day 0 Day 5 Day 10 P^‡^	36.30 ± 3.62 29.95 ± 3.14 19.37 ± 2.73 **0.001**	41.03 ± 4.49 38.09 ± 3.67 32.78 ± 3.03 0.211	0.329^¥^ 0.156^£^ 0.018^£^
IL-22	Day 0 Day 5 Day 10 P^‡^	186.30 ± 22.15 164.75 ± 14.88 109.37 ± 10.03 ** < 0.0001**	201.03 ± 20.52 190.09 ± 16.03 177.78 ± 15.68 0.409	0.403^¥^ 0.211^£^ 0.004^£^

Data are presented as means ± SD; Numbers in bold are statistically significant *p*-value < 0.05.

HMGB: High mobility group box 1; IL: interleukin.

^¥^Based on independent sample *t*-test.

^£^Based on analysis of covariance (ANCOVA); adjusted for sex, age, type of disease and baseline values.

^‡^Based on Repeated-measure analysis of variance.

### Effect of nano curcumin on forkhead box P3, NLR family pyrin domain containing 3, interferon gamma, and nuclear factor kappa B genes expression

**FIGURE 2:**
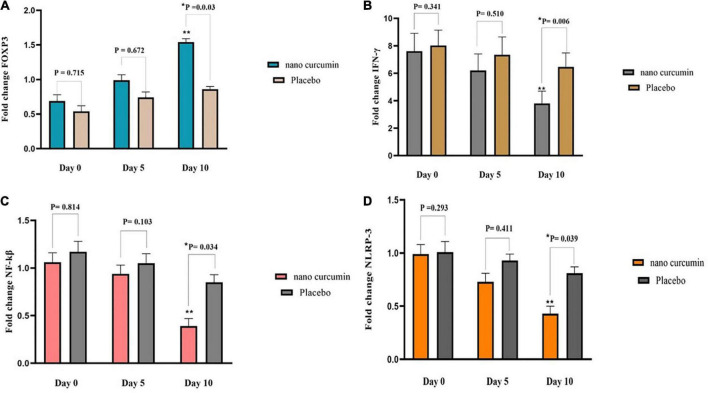
The effects of the intervention on FOXP3, NLRP3, IFN-γ, and NF-kβ expression in two study groups. **(A)** Fold change of FOXP3 **(B)** fold change of NLRP3 **(C)** fold change of IFN-γ **(D)** fold change of NF-kβ. Values are mean of fold change ± SEM. Data analysis was done using the ANCOVA test (adjusted for sex, age, type of disease, and baseline values; *p < 0.05 vs. placebo) and Repeated measures ANOVA (**p < 0.05 vs. baseline). *P* < 0.05, statistically significant. FOXP3, forkhead box P3; NF-κB, nuclear factor kappa B; NLRP3, NLR family pyrin domain containing 3; IFN-γ, interferon gamma.

Forkhead box P3 (FOXP3), NLR family pyrin domain containing 3 (NLRP-3), interferon gamma (IFN-γ), and nuclear factor kappa B (NF-kβ) levels did not change substantially after 5 days of supplementation with NC, as shown in Figure 2. In the NC group, FOXP3 expression considerably increased compared to the placebo group, after 10 days (fold change: 2.69 ± 0.99 vs. 3.24 ± 0.74 (*P* = 0.022) (Figure 2A). At the end of the study, NC supplementation significantly reduced mRNA expression of IFN-γ (fold change: 0.39 ± 0.11) compared to placebo (fold change: 1.26 ± 0.07). (*P* = 0.006) (Figure 2B). Also, on the 10th day, the NC group’s NF-kβ expression level (fold change: 1.13 ± 0.11) was higher than the placebo group’s fold change: 1.51 ± 0.07) (*P* = 0.014) (Figure 2C). Moreover, NC supplementation led to a significant decrease in NLRP-3 mRNA expression (fold change: 2.07 ± 0.79) than placebo (fold change: 2.99 ± 0.46), (*P* = 0.039) (Figure 2D).

### Effect of nano curcumin on Clinical outcomes

In Table 7, effect of curcumin on clinical outcomes are presented for the participants. At the end of the study, the NC group’s MODS and SOFA scores decreased significantly compared to the placebo group (*P* < 0.05). Furthermore, there was no remarkable alteration between the two groups in the length of stay in the ICU, systolic blood pressure, dystopic blood pressure saturation (%), respiratory rate (breaths/min) PaO2/FiO2.

**TABLE 7:** Effect of curcumin on clinical outcomes are presented for the participants.

Variables		Nano-curcumin group (*n* = 20)	Placebo group (*n* = 20)	*Pv*
Length of hospital stay (day)		27.40 ± 6.18	32.10 ± 9.17	0.083^¥^
SOFA score	Day 0 Day 5 Day 10 P ^T–^	8.10 (5–11) 7.55 (5–9) 6.1 (7–4) **0.0001**	7.50 (6–11) 6.95 (6–10) 6.7 (5–8) 0.120	0.538^†^ 0.207^£^ **0.027 ^£^**
MODS score	Day 0 Day 5 Day 10 P ^T–^	9.14 (6–15) 7.54 (6–12) 5.04 (5–8) **0.0015**	10.06 (8–15) 8.95 (7–13) 7.01 (5–9) 0.060	0.715^†^ 0.346^£^ **0.025 ^£^**
DBP (mmHg)	Day 0 Day 5 Day 10 P ^T–^	9.98 (8–11) 9.21 (8–10) 8.16 (7–9) **0.036**	9.71 (8–11) 9.15 (8–10) 8.2 (7–9) 0.054	0.641^†^ 0.417^£^ 0.136 ^£^
SBP (mmHg)	Day 0 Day 5 Day 10 P ^T–^	15.21 (14–18) 13.80 (13–17) 11.63 (12–14) **0.0032**	16.06 (14–19) 14.89 (13–17) 12.94 (12–14) **0.0045**	0.541^†^ 0.892^£^ 0.125 **^£^**
PaO2/FiO2 (mmHg)	Day 0 Day 5 Day 10 P^‡^	176.50 ± 21.32 155.83 ± 17.40 123.10 ± 20.74 **0.003**	183.42 ± 23.01 166.77 ± 16.49 137.62 ± 18.28 **0.031**	0.439^¥^ 0.241 ^£^ 0.077^£^
Respiratory rate (breaths/min)	Day 0 Day 5 Day 10 P^‡^	18.21 ± 2.84 16.98 ± 2.76 14.85 ± 2.32 0.061	19.12 ± 2.20 17.16 ± 2.59 15.21 ± 2.73 0.114	0.518^¥^ 0.414 ^£^ 0.097^£^

ICU, intensive care unit; SOFA, Sequential Organ Failure Assessment, MODS: Multiple Organ Dysfunction Score; SBP: systolic blood pressure DBP: diastolic blood pressure; partial pressure (PaO2 in mmHg) to fractional inspired oxygen (FiO2).

Data are presented as means ± SD; Numbers in bold are statistically significant *p*-value < 0.05.

^£^Based on analysis of covariance (ANCOVA) after logarithmically converting; adjusted for sex, BMI, age, type of disease and baseline values.

^¥^Based on independent sample *t*-test.

^†^Based on Mann–Whitney U Test.

## Discussion

The present RCT evaluated the impacts of NC supplementation on immune response in septic patients admitted to ICU. The current study revealed that 10 days of NC supplementation could significantly reduce IL-17, IL-22, SOFA, and MODS scores serum levels. It also decreased the mRNA expression of NLRP-3, NF-êB, HMGB-1, and IFN-γ genes and increased the mRNA expression of FOXP3. As far as we are aware, this is the first study to evaluate NC’s effect on immune response in patients with sepsis. Sepsis, which is defined as the excessive activity of the immune system in dealing with pathogenic factors, leads to systemic inflammatory response, coagulation disorders, endothelial function, and immune response (28), and it is a major cause of death in ICUs (29). We found that NC supplementation significantly decreased serum concentrations of IL-17 and IL-22 in septic patients after 10 days of intervention. Various studies have assessed the effect of curcumin on pro-inflammatory cytokines. A study conducted by Silva et al. (30) showed that treatment of septic rats with 100 mg/kg of curcumin remarkably lessened the pro-inflammatory cytokines, namely IL-1β and IL-6. In another study, Djalali et al. (31) reported that 2 months of NC supplementation declined the serum concentration of IL-17 and its mRNA expression in patients with episodic migraine. There is growing evidence that high concentrations of IL-17 are correlated to a higher peril of sepsis, which could provide a biomarker for the prognosis of sepsis (32). IL-17 interacts with various mediators, namely IL-1β, TNF-α, and IL-22 to exert its pro-inflammatory impact (33). Also, IL-22 plays a pivotal role in chronic inflammatory diseases and polymicrobial sepsis (34). In a trial conducted by Antiga et al. (27), curcumin (2g/day) supplementation considerably decreased the serum levels of IL-22 in participants with mild-to-moderate psoriasis Vulgaris. However, unlike the results of our study, curcumin did not significantly affect the serum level of IL-17 (27). This contrary finding might be due to the different underlying diseases of the participants and the distinct forms of curcumin used in the trials.

The inflammatory responses during sepsis might lead to the dysfunction of vital organs, including the lung, kidneys, heart, and liver, and thus, cause MODS (28). The number of organs engaged in MODS is positively correlated to the mortality of sepsis (35). Scores such as SOFA and MODS are used to properly identify septic patients at higher risk of mortality (29). In the present study, although NC supplementation did not affect the respiratory rate, PaO2/FiO2, and blood pressure, it significantly decreased the SOFA and MODS scores after 10 days. This finding is in line with the results of several animal studies (36, 37). Chen et al. (36) presented that supplementation with curcumin diminished tissue injury and improved survival rates in septic mice. Moreover, the results of another experimental study indicated that curcumin could prevent dysfunction of the kidneys, liver, and small bowel in rats with experimentally formed sepsis (37).

Additionally, the present study showed that septic patients who received NC for 10 days had significantly lower levels of NLRP-3. This finding was in agreement with an animal study that reported the suppression of NLRP-3 inflammasome activation in mice treated with a curcumin analog (38). Moreover, Gong et al. (39) indicated that curcumin could decrease the level of IL-1β by inhibiting the activation of NLRP-3 in an *in vivo* study. On the contrary, 12 weeks of curcumin supplementation among hemodialysis patients did not substantially influence NLRP-3 mRNA expression (40). This might be explained by different study sample sizes, carriers of the curcumin, as well as different participants of the studies. NLRP3 is a major component of the innate immune system that is prompted by pathogens and releases pro-inflammatory cytokines (41, 42).

**FIGURE 3:**
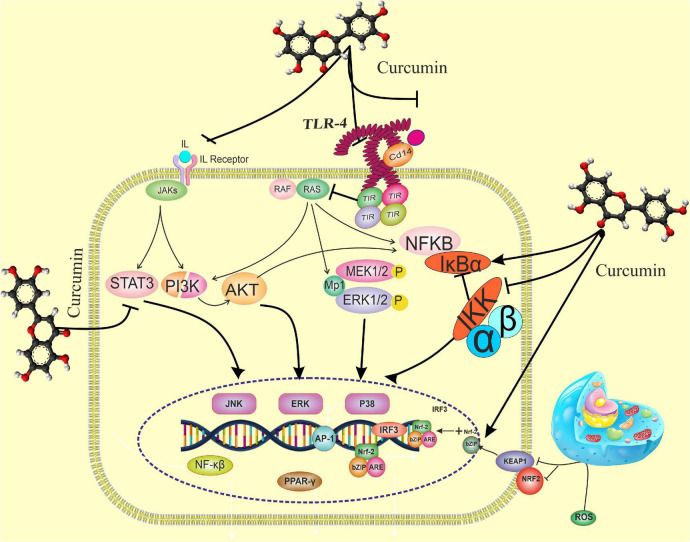
The effects of curcumin on the immune response pathway. Curcumin can bind directly to MD2 (protein appears to connect with toll-like receptor 4 on the cell surface). Curcumin also inhibits LPS-induced activation of MyD88- and TRIF-dependent TLR4 pathways, resulting in suppression of both IRF3 and NF-B. Curcumin promotes the expression of the Nrf-2 gene by boosting the antioxidant capacity and synthesis of antioxidant enzymes such as SOD, GPX CAT, and CAT. TANK-binding kinase 1; TRAF, TNF receptor-associated factor; TRAM, TRIF-related adaptor molecule; TRIF, TIR-domain- containing adapter-inducing interferon-β, Nrf-2, nuclear factor erythroid 2-related factor 2; p-IκBα, phosphorylated-IκBα; s; ROS, reactive oxygen species, STAT1, signal transducer and activator of transcription 1; TGF-1β, transforming growth factor-β1; TIRAP, toll-interleukin 1 receptor domain-containing adaptor protein; TLR4, toll-like receptor 4; TNF-α, tumor necrosis factor-α.

Additionally, NC significantly reduced NF-kB expression in septic patients. In an animal study (43), both treatment and pretreatment with curcumin lessened NF-êB activation in renal tissues of septic rats. Xie et al. (44) also revealed that curcumin exerts its protective effects on lipopolysaccharide (LPS)/D-galactosamine (D-GalN)-induced acute liver injury in rats by up-regulating nuclear Nrf-2 and downregulate NF-êB. The activated NF-êB is a chief regulator of inflammatory gene expression, including NLRP-3 (Figure 3) (45).

Several mechanisms have been suggested for the effects of curcumin administration on the components of the immune response mentioned above. Curcumin can down-regulate the Th1 and Th17 cell pathways and help modulate T-helper immune responses (27). It is speculated that curcumin adjusts the Treg/Th17 rebalance by inhibiting the IL-23/Th17 pathway (46). Another hypothesis is that curcumin down-regulates the expression of IL-22 and IL-17 indirectly by repressing IL-1β and IL-6 due to their synergistic activities with IL-17 (47). In addition, curcumin blocks the phosphorylation and degradation of IêB, the inhibitor protein of NF-êB, and averts the nuclear translocation of NF-êB (30). By inhibiting the activation of NF-êB, the transcription of genes engaged in the expression of pro-inflammatory cytokines is suppressed (48). Additionally, curcumin increases the expression of peroxisome proliferator−activated receptor gamma (PPARγ), which contributes to the suppression of NF-êB and lowers the release of pro-inflammatory cytokines (49).

The inhibition of the NF-êB pathway can alleviate the severity of MODS (50). Also, myeloperoxidase which represents polymorphonucleocytes infiltration, is a major indicator of tissue damage (50). By blocking myeloperoxidase activity, curcumin decreases tissue injury (37, 50). Curcumin’s anti-inflammatory and antioxidant properties generally reduce organ dysfunction during sepsis (36, 37, 50).

The important role of NF-κB activation is the regulation of NLRP3, ASC, and caspase-1 gene expression as well as the production of pro-inflammatory cytokines (51, 52). Thus, curcumin inhibits NF-êB signaling and suppresses NLRP-3 activation (53). Besides, curcumin attaches to peroxiredoxin 1 (PRDX1), which interacts with pro-caspase-1 and suppresses the link between pro-caspase-1 and ASC; therefore, the assembly of NLRP-3 inflammasome discontinues (38).

The mRNA expression level of HMGB1 was considerably reduced after 10 days of NC supplementation in patients with sepsis. During sepsis, high concentrations of HMGB-1 stimulate the production of pro-inflammatory cytokines, which are associated with MOD and mortality (54, 55). In accordance with our study, Ahn et al. (56) reported inhibiting the production of HMGB1 in endotoxemia mice with curcumin longa extract-loaded nanoemulsion. In addition, in a study by Kim et al. (57), curcumin inhibited the LPS-mediated release of HMGB-1 by endothelial cells and down-regulated the expression of HMGB-1 receptors. The proposed mechanism for the effect of curcumin on HMGB-1 is the blocking of nitric oxide due to the suppression of c-Jun N-terminal kinase, which leads to the inhibited release of HMGB-1 by macrophages (56).

Another finding of the current study was that mRNA expression of IFN-γ was considerably reduced after 10 days of NC supplementation. An experimental study by Gao et al. (58) indicated that curcumin treatment markedly suppressed the IFN-γ gene expression by splenic T lymphocytes. In addition, Kang et al. (59) demonstrated that in macrophages stimulated with LPS or heat-killed *Listeria monocytogenes*, pretreatment with curcumin decreased the production of IFN-γ. When concentrations of IFN-γ exceed a particular level in sepsis, resistance to infections is impaired, leading to an increased lethality rate (60, 61). Therefore, suppressing IFN-γ to normal levels is beneficial to the host due to preventing bacterial outflow (62). The mechanism by which curcumin reduces IFN-γ is that curcumin decreases CD4^+^ and IFN-γ^+^ and thereby inhibits the Th1 response (63). In addition, curcumin inhibits the Th1 cytokine profile by inhibiting IL-12 production (59).

The other immune response factor that we assessed in the current study was FOXP3. The results showed that NC supplementation considerably increased the mRNA expression of FOXP3. In line with our finding, Chen et al. (36) reported that curcumin administration elevates the expression of FOXP3 in septic mice compared to mice treated with corn oil. FOXP3, a key regulator of T regulatory (Treg) cell development and function, is expressed on CD4^+^ CD25^+^ Treg cells (64, 65). In a study by Chai et al. (66), curcumin attenuated the acute lung injury of cecal ligation and puncture-induced mouse model by boosting the differentiation of naïve CD4^+^ T cells to CD4^+^ CD25^+^ FOXP3^+^ Tregs. Regarding the mechanism underlying the effect of curcumin on FOXP-3, it is suggested that curcumin increases the CD4^+^, CD25^+^, and FOXP3^+^ Treg cells (36), which in turn increases the expression of anti-inflammatory cytokine IL-10 and decreases the proliferation activity of CD4^+^, CD25^–^, and T cells (36, 67).

So far as we are aware, no previous research has assessed the effect of NC on immune response among septic patients in ICU. In addition, randomizing the participants minimized the possibility of confounding factors. However, this study is not without limitations. First, the results cannot be generalized since we did not include refractory septic shock patients. Second, a longer supplementation duration and higher NC doses might lead to greater efficacy.

In conclusion, our results indicated that NC supplementation for 10 days in ICU patients with sepsis significantly decreased pro-inflammatory cytokines, MODS, and SOFA scores, the mRNA expression of NF-êB, NLRP-3, IFN-γ, and increased the expression of FOXP3. Further trials with a longer intervention period and larger sample size are warranted to confirm these findings.

## Data availability statement

The original contributions presented in this study are included in the article/supplementary material, further inquiries can be directed to the corresponding author.

## Ethics statement

The studies involving human participants were reviewed and approved by Tabriz University of Medical Sciences (IR.TBZMED.REC.1396.762). The patients/participants provided their written informed consent to participate in this study.

## Author contributions

AK, SP, MV, AM, SS, and MK designed the first hypothesis of the work and searched the data. AK and MV read and extracted the data. AK and AT-E wrote the draft of the manuscript. All authors have read and approved the final manuscript.
